# Corticosteroid Receptors in the Brain: Transcriptional Mechanisms for Specificity and Context-Dependent Effects

**DOI:** 10.1007/s10571-018-0625-2

**Published:** 2018-10-05

**Authors:** Onno C. Meijer, J. C. Buurstede, Marcel J. M. Schaaf

**Affiliations:** 1 Division of Endocrinology, Department of Medicine, Leiden University Medical Center, 2333 ZA Leiden, The Netherlands; 2 Einthoven Laboratory for Experimental Vascular Medicine, Leiden University Medical Center, 2333 ZA Leiden, The Netherlands; 3 Department of Animal Sciences and Health (M.J.M.S.), Institute of Biology, Leiden University, 2333 CC Leiden, The Netherlands

**Keywords:** Glucocorticoids, Brain, Stress, Transcription, Hippocampus

## Abstract

Corticosteroid hormones act in the brain to support adaptation to stress via binding to mineralocorticoid and glucocorticoid receptors (MR and GR). These receptors act in large measure as transcription factors. Corticosteroid effects can be highly divergent, depending on the receptor type, but also on brain region, cell type, and physiological context. These differences ultimately depend on differential interactions of MR and GR with other proteins, which determine ligand binding, nuclear translocation, and transcriptional activities. In this review, we discuss established and potential mechanisms that confer receptor and cell type-specific effects of the MR and GR-mediated transcriptional effects in the brain.

## Introduction

Corticosteroids are potent modulators of neurons and non-neuronal brain cells. In humans, cortisol is the main corticosteroid hormone that is secreted upon stress, whereas in rodents corticosterone plays this role. Two corticosteroid receptors mediate the vast majority of the effects of cortisol and corticosterone: high-affinity mineralocorticoid receptors (MRs) and lower-affinity glucocorticoid receptors (GRs). MR and GR action as transcription factors is thought to underlie many responses to cortisol in a time frame that spans from hours (Oitzl and Ronald de Kloet 1992) to weeks or even years. Fascinating studies on pain sensitivity showed that glucocorticoids can act as a switch, to instate long-term changes in pain sensitivity: the effects of removing the adrenals from rats differed dramatically depending on the acute levels of corticosterone at the moment of the operation (Ratka et al. 1988; Marinelli et al. 1997). Long-term exposure to cortisol in Cushing’s patients is manifested 10 years after normalization of hormone levels as disturbances in brain gray and white matter organization (Andela et al. 2013; van der Werff et al. 2014). Non-genomic effects have also been reported and mediate more rapid responses (Gutièrrez-Mecinas et al. 2011; Joëls et al. 2013; Gasser and Lowry 2018). The transcriptional activity of corticosteroid receptors is the focus of the present review article, and we will discuss how MR and GR specificity in response to ligand binding may be brought about.

Transcriptional regulation is intrinsically context dependent (Weikum et al. 2017). Indeed, there is a pronounced cell type/regional specificity of brain corticosteroid effects, exemplified by the opposite directionality of GR-mediated *Crh* gene expression in hypothalamus and amygdala (Kolber et al. 2008) (Makino et al. 1994) (Zalachoras et al. 2016), and by the opposite effects of corticosteroids on dendritic complexity in distinct brain regions and circuits (Magariños and McEwen 1995; Mitra and Sapolsky 2008; Dias-Ferreira et al. 2009). This regional specificity is partially explained by different localization of the receptors (Reul and De Kloet 1985), but mostly reflects cell type-specific differences in chromatin structure and different subsets of transcriptionally active proteins that interact with MR and GR.

MR and GR can mediate very different, and sometimes opposite effects in the brain that range from effects on neurotransmitter responsiveness at the level of a single hippocampal neuron (Joëls et al. 1991), to their protective/endangering effects for psychopathologies (Spijker et al. 2009; Klok et al. 2011b). On the other hand, MR and GR share a number of canonical target genes, and apparently can have very similar effects on the regulation of such genes (D’Adamio et al. 1997; Robert-Nicoud et al. 2001).

### Receptor Structure

**Fig. 1:**
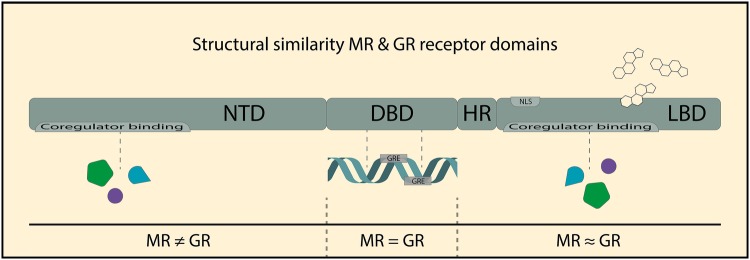
Structure and functional domains of the mineralocorticoid receptor (MR) and glucocorticoid receptor (GR). MR and GR belong to the nuclear receptor super family and comprised an N-terminal domain (NTD), a DNA-binding domain (DBD), a hinge region (HR), and a ligand-binding domain (LBD). Ligands are bound by the generally well-conserved LBD, leading to a set of ligands (e.g., cortisol) which can activate both the MR and GR. The LBD also contains a nuclear localisation signal (NLS), which is important for translocation to the nucleus after ligand binding. The highly conserved DBD enables DNA binding of both the MR and GR to a glucocorticoid response element (GRE) on the DNA. Coregulator proteins that can modulate the transcriptional output can bind to both the LBD and the NTD, of which the latter is the most divergent domain between the MR and GR

MR and GR are both members of the nuclear receptor family of transcription factors, with the typical modular protein structure of a central DNA-binding domain (DBD), a C-terminal ligand-binding domain (LBD), and an N-terminal domain (Oakley and Cidlowski 2013) (Fig. 1). The LBD and especially the DBD show a high level of evolutionary conservation between the two receptors. The basic function of the different domains is reasonably well understood, and discussed in more detail below. It is important to note that there are many post-translational modifications of the receptors, that can substantially alter the activity of the receptors (Lambert et al. 2013). These will not be extensively described here, but have been reviewed recently (Vandevyver et al. 2014; Faresse 2014; Kino 2018).

The LBD contains the ligand-binding pocket that determines binding specificity and affinity. It also contains the amino acid residues that are responsible for the initiation of conformational changes upon binding of ligands, which subsequently leads to receptor translocation to the nucleus and enables interactions with DNA and transcription-related proteins in the cell nucleus. The LBD also harbors one of the two transcriptional output domains: the ‘AF-2’ protein surface that in a ligand-dependent manner interacts with downstream proteins that mediate the transcriptional effects of the receptors (Vandevyver et al. 2014; Starick et al. 2015).

The DBD is 96% identical between MR and GR. Via the DBD, both receptors bind to glucocorticoid response elements (GREs) in the DNA, which consist of two half sites, each of which serve as a docking site for one receptor, leading to dimer formation on a full GRE. Mutations in the DBD dimer interface impair receptor binding (Liu et al. 1995; Reichardt et al. 1998), but there are GRE-binding sites that are independent of classical receptor dimerization (Adams et al. 2003; Lim et al. 2015). The high similarity between MR and GR DBDs allows for heterodimerization on the same GREs, which has been observed in neuronal cells (Trapp and Holsboer 1996; Mifsud and Reul 2016; Weikum et al. 2017). Recent studies suggest that also higher-order receptor complexes, i.e., tetramers, may occur at the GRE (Kolber et al. 2008; Presman and Hager 2017). The DBD is not just a rigid docking domain: DBD interaction with the nucleotides of the GRE can modify the exact conformation of the receptor, and in this way the GRE allosterically modulates the transcriptional activity of the receptor (Meijsing et al. 2009). Regardless of the exact make-up of the receptor complexes on the DNA, direct binding of MR and GR to GREs seems to be the dominant mode of signaling for both transcription factors in the brain (Polman et al. 2013; van Weert et al. 2017; Pooley et al. 2017).

The N-terminal domain differs substantially in amino acid content between MR and GR. This domain is ‘intrinsically unstructured,’ meaning that it will adopt a particular conformation depending on molecular partners (Kumar and Thompson 2012). This domain is important in that it contains the AF-1 region that is able to stimulate transcription even in the absence of ligand. Of note, the N-terminal domain can differ in length, as a consequence of alternative translation initiation (Lu and Cidlowski 2005; Viengchareun et al. 2007), and these translation variants may differ in their activities (Wu et al. 2013; Oakley et al. 2018). Because it is difficult to distinguish between these protein variants in vivo (other than with antibodies that recognize the actual N-terminus), their relevance for brain function remains largely unknown.

### Splice Variants and Alternative Promoter Activity

From the genes encoding GR and MR (*Nr3c1* and *Nr3c2*), differentially spliced mRNAs can be generated resulting in the occurrence of receptor variants. Variation in the C-terminal part of the receptor is most common, with the human GR beta as the best known example (van der Vaart and Schaaf 2009). hGRbeta does not bind cortisol, and may be a cause of glucocorticoid resistance (Oakley and Cidlowski 2013). However, this receptor isoform was not detected in the human brain at appreciable levels, at least under non-inflammatory conditions (Derijk et al. 2003). Also other splice variants within the coding region of the genes exist (Zennaro et al. 2001; Oakley and Cidlowski 2013). In addition, MR and in particular GR vary in their non-coding first exon usage. This reflects the activity of alternative promoters and has no consequences for the structure of the encoded receptor protein (Turner et al. 2006; Klok et al. 2011a). The GR intron 1F (1–7 in rat and mouse) has received considerable attention, based on its differential CpG methylation as a consequence of early-life stress/trauma (Liu et al. 1997; McCormick et al. 2000). It is of note that this intron is responsible for only a fraction of the total amount of GR mRNA, and its methylation should be seen as a marker for broader methylation of the gene (Weaver et al. 2006; Alt et al. 2010; Witzmann et al. 2012).

### Receptor Localization

MR and GR activation clearly lead to different effects on brain function. The most direct way to achieve receptor-specific effects is by differential receptor expression per brain region. It is known since 1985 that indeed GR and MR differ in their brain expression patterns (Reul and De Kloet 1985). GR is expressed at varying degrees throughout the mouse brain—with the interesting exception of the suprachiasmatic nucleus (Rosenfeld et al. 1998; Balsalobre et al. 2000). MR expression on the other hand is much more restricted. It is very abundant in the rodent and human hippocampus where MR expression equals or exceeds that of GR in CA3 pyramidal cells (Mahfouz et al. 2016). In addition, it is present in other limbic brain areas such as the prefrontal cortex and amygdala (Venkova et al. 2009; McEown and Treit 2011; Qi et al. 2013).

MR and GR are expressed in neuronal cells and there is compelling evidence for direct corticosteroid effects on specific types of neurons via both MR and GR (Joëls 1997; Ambroggi et al. 2009; Hartmann et al. 2016). In addition, GR expression has been demonstrated in oligodendrocytes (van Gemert et al. 2006), astroglia (Koyanagi et al. 2016; Piechota et al. 2017), and microglia (Tentillier et al. 2016). Moreover, it has been argued that MRs and GRs localized in the brain vasculature should be taken into account when considering brain processes (Gomez-Sanchez 2014). Here it is relevant that MRs in endothelial and vascular smooth muscle cells may be responsive to aldosterone, rather than cortisol. This is a consequence of the expression of the enzyme 11β-hydroxysteroid dehydrogenase type 2 that inactivates endogenous corticosteroids to their 11-dehydro metabolites (like cortisone in the case of cortisol) (Bender et al. 2013).

## Mode of Action

### Receptor Activation and Nuclear Translocation

In the absence of hormone, MR and GR reside in the cytoplasm in a multiprotein complex. This consists of an Hsp90 dimer, p23, and one of the tetratricopeptide repeat (TPR)-containing co-chaperones: the immunophilin FK506-binding proteins (FKBP) 51 or 52, cyclophilin 40 (CyP40), or protein phosphatase 5 (PP5). The assembly of this complex is ATP-driven and requires the involvement of Hsp70, Hop, and Aha1 (Ratajczak 2015). The incorporation of FKBP51 in a complex with GR results in decreased ligand affinity of the receptor and reduced nuclear translocation efficiency (Wochnik et al. 2005). As a result, GR-induced upregulation of FKBP51 expression acts as a negative feedback mechanism to decrease glucocorticoid sensitivity (Davies et al. 2005; Banerjee et al. 2008; Cluning et al. 2013). This mechanism has been linked to the pathogenesis of psychiatric diseases, as a result of epigenetic alterations in the GRE that mediates FKBP51 induction (Klengel et al. 2013). The FKBP51 gene may also be regulated via MR, as it contains a GRE that is bound by both MR and GR in vivo (Mifsud and Reul 2016). Upon ligand binding, switching from a complex with FKBP51 to one including FKBP52 is a first step in GR activation and results in recruitment of dynein and nuclear translocation of the receptor (Davies et al. 2002). The dissociation of the phosphatase PP5 from the complex upon ligand binding contributes to receptor phosphorylation which modulates the transcriptional activity in a gene-dependent manner (Wang et al. 2007). Similar effects of these specific co-chaperones and ligand-induced changes in the composition of the multiprotein receptor complex have been demonstrated for MR (Gallo et al. 2007).

MRs and GRs are known to shuttle between the cytoplasmic and nuclear compartments and the subcellular distribution of MR and GR depends on the equilibrium between nuclear import and export. This equilibrium appears to be cell type specific, since immunofluorescent analysis in the rat hippocampus shows that in adrenalectomized rats, substantial nuclear localization of MR and GR was observed in dentate gyrus, but not in CA1 pyramidal cells (Sarabdjitsingh et al. 2009). Nuclear import of MR and GR starts with transport of the multiprotein receptor complex towards the nucleus along microtubules, which is followed by passage of the entire complex through the nuclear pore complex (NPC). Both steps require the presence of Hsp90, FKBP52, and dynein in the multiprotein complex, and dissociation of the receptors from this complex occurs in the nucleoplasm (Galigniana et al. 2010). Reassociation of GR with components of this complex like p23 and hsp90 has been shown to disrupt the transcriptional activity of the receptor (Freeman and Yamamoto 2002). A role for the microtubule-associated protein doublecortin-like in the transport of GR was demonstrated in neuronal progenitor cells (Fitzsimons et al. 2008). When the cytoskeleton is disrupted, the receptors may move to the NPC by passive diffusion. Nuclear import through the NPC is dependent on nuclear localization signals (NLSs). GR contains two NLSs (Picard and Yamamoto 1987), whereas MR contains three (Walther et al. 2005). In both cases, the function of the most C-terminal NLS, located in the LBD, is dependent on ligand binding. The NLS sequences are bound by importins, which translocate to the nucleus through the NPC. Importins 7, 8, 13, and α/β are known to be involved in GR nuclear translocation. Once in the nucleus, the importins dissociate from the receptor by binding to RanGTP. Nuclear export of MR and GR follows a similar mechanism in the opposite direction. The exportin Calreticulin binds in a Ca^2+^-dependent way to a nuclear export signal (NES), which is located between the two zinc fingers in the DBD. This complex associates with RanGTP and is transported out of the nucleus through the NPC.

### Finding Target Sites Inside the Nucleus

Inside the nucleus, MRs and GRs interact with specific DNA target sites to regulate transcription. How the receptors reach these sites in the vast amount of DNA is starting to become clear. The advancement of fluorescence microscopy techniques in recent years has enabled detailed analysis of the mobility pattern of fluorescently labeled proteins (Mueller et al. 2013; van Royen et al. 2011). Studies, in which fluorescence recovery after photobleaching (FRAP) and single-molecule microscopy were combined, have shown that the mobility of MR and GR inside the nucleus is very similar (Groeneweg et al. 2014). Recently, we have demonstrated that four different states of GR molecules inside the nucleus can be distinguished based on their mobility (Keizer, Schaaf et al., unpublished). In two of these states, receptors are diffusing through the nucleus, with either a diffusion coefficient of 3.1 µm^2^/s, or with an approximately sixfold lower diffusion coefficient. In the other two states, the receptors are immobile, with an immobilization time of approximately 0.5 or 3 s, and the exact times are dependent on the ligand. Based on functional studies, the fast diffusing population was interpreted as receptors freely diffusing through the nucleus (possibly in a complex with chaperones), and the slowly diffusing population as receptors of which the diffusion is interrupted by random brief (< 1 ms) interactions with DNA. The longer immobilizations were interpreted as (direct and indirect) binding to specific DNA target sites. The results of our analysis further showed that GRs spend a relatively long time in the free diffusion state (more than 7.5 s on average). When they leave this state, they almost always transit to the slow diffusion state. Interestingly, from this slow diffusing state they almost always go to one of the immobile states and subsequently they alternate between these states for a long period of time before returning to the free diffusion state. This process of alternating between the slow diffusion and immobile state has been called the ‘repetitive switching’ mode.

### Chromatin Interaction

**Fig. 2:**
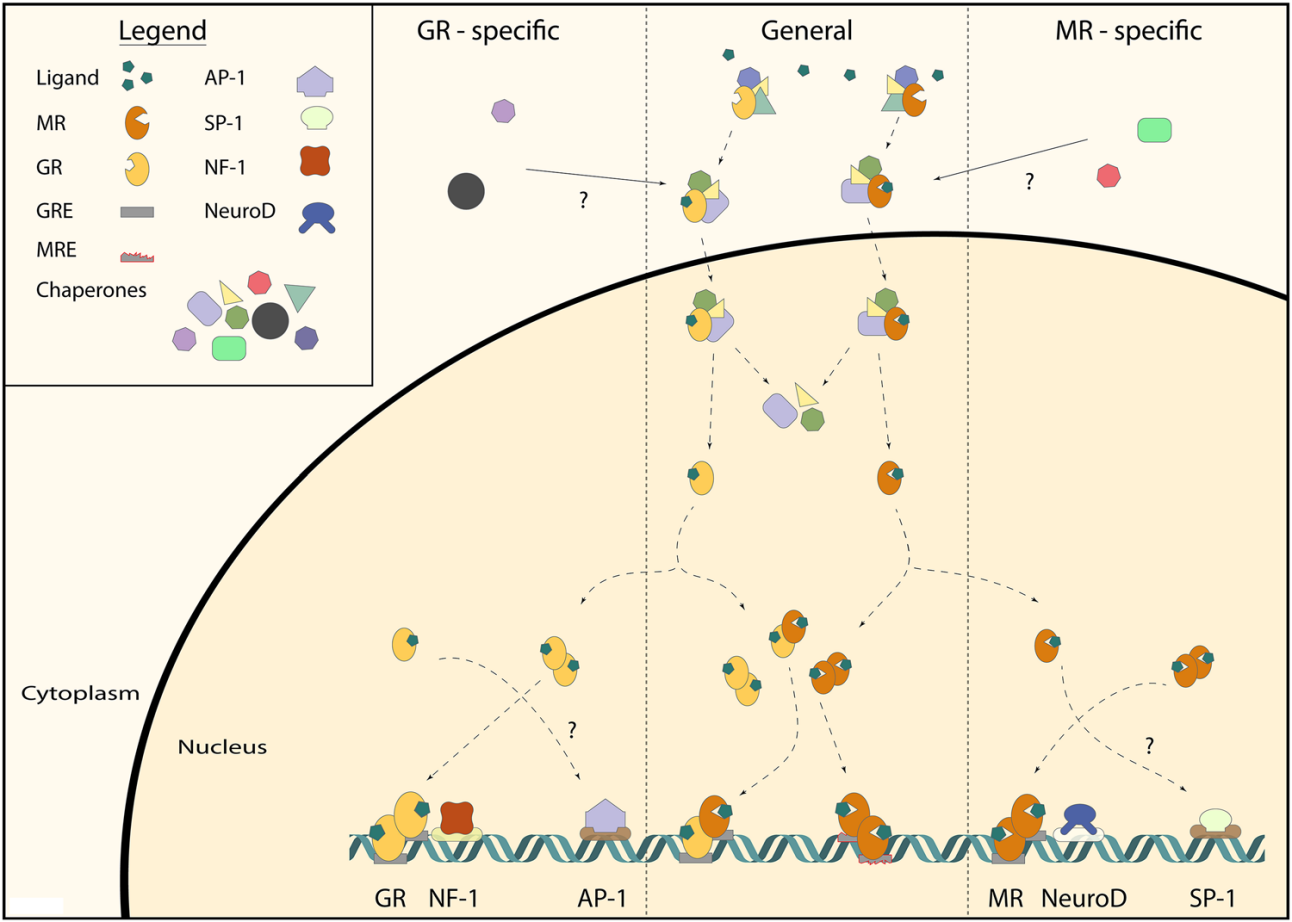
General and receptor-specific interactions underlying MR and GR signaling. MRs and GRs reside in the cells cytoplasm bound by chaperone proteins (e.g., Hsp90, Fkbp5). The existence of receptor-specific chaperone proteins is unclear (top). Upon ligand binding, this multiprotein complex translocates to the nucleus, where it dissociates. Subsequently, the MR and GR can form either homo- or heterodimers or function as monomers. MR and GR homo- and heterodimers bind to the glucocorticoid response elements (GRE) in order to exert their genomic effects. MR and GR-specific binding are mainly linked to co-occurrence with binding motifs of other transcription factors (e.g., NF-1 for GR and NeuroD for leading to an actual ‘MRE’) indicating interactions between these transcription factors. GRE-independent, receptor-specific monomer interactions with other transcription factors (e.g., AP-1 for GR and SP-1 for MR) are known in other tissues, but the presence of these is not yet shown in the brain

MR and GR have two major modes of DNA binding: directly to (variations of) GREs, and indirectly by ‘tethering’ to other transcription factors (Fig. 2). The anti-inflammatory effects of corticosteroids are mediated by GR, and these depend for a substantial part on GR’s inhibitory binding as monomers to proinflammatory transcription factors, such as AP-1 and NF-kB. MR is much weaker in transrepressing AP-1 (Pearce and Yamamoto 1993), but has been shown to transrepress the transcription factor SP1 (Meijer et al. 2000b; Meinel et al. 2013). Classically direct DNA binding is considered as stimulating gene expression, and tethering as repressing this process. However, more recent evidence shows that tethering may also have stimulating effects. For example, positive interactions between GR and the transcription factor Stat3 occur when Stat3 interacts with DNA-bound GR (Langlais et al. 2012). Moreover, negative GREs exist and are prominent in the core of the HPA-axis, where they mediate GR-dependent repression of *Crh* and *Pomc* genes in the hypothalamus and pituitary corticotrophs, respectively. These negative GREs are however much more widespread, including an intronic nGRE that contributes to homologous downregulation of GR (Surjit et al. 2011; Ramamoorthy and Cidlowski 2013; Oakley and Cidlowski 2013).

Genome-wide studies of MR and GR binding to DNA have been performed using chromatin immunoprecipitation (ChIP)-sequencing analysis. Subsequent motif analysis of MR- and/or GR-bound DNA loci has yielded information on whether there are GRE-like sequences present, or rather binding sites of potential tethering partners. Analysis of MR DNA binding in a human kidney cell line showed that, although a GRE sequence was the most prevalent motif among MR binding sites, the majority of MR binding sites involved indirect DNA binding through interaction with other transcription factors (FOX, EGR1, AP1, PAX5) (Le Billan et al. 2015). Similar data have been found for GR binding in many tissues (John et al. 2011; Rao et al. 2011; Uhlenhaut et al. 2012). In contrast, three ChIP-seq datasets from rat hippocampal tissue are available, and they suggest that in this brain region both MR and GR act predominantly via direct binding to GRE sequences (Polman et al. 2013; van Weert et al. 2017; Pooley et al. 2017).

Interestingly, often times other DNA motifs were present in the vicinity of the GREs (Polman et al. 2013; van Weert et al. 2017; Pooley et al. 2017). A comparison of GRE sequences around proven corticosterone target genes in the rat hippocampus revealed a number of strong evolutionary conserved GRE sequences around these genes (Datson et al. 2013). However, actual GR binding occurred at only 50% of these GREs, and these functional sequences were enriched in binding motifs for transcription factors like MAZ1 (Datson et al. 2011). Later, genome-wide binding studies identified NF-1 motifs being present in 50% of the loci that were bound by GR. A binding motif for transcription factors of the NeuroD family occurred in about 15% of the GR binding loci (Pooley et al. 2017).

MR and GR can regulate transcription by binding to the same GRE sequences, as is clear for genes like *Per1, Fkbp5, Gilz, and Sgk1* (Webster et al. 1993; D’Adamio et al. 1997; Chen et al. 1999; Robert-Nicoud et al. 2001; Mifsud and Reul 2016). However, MR and GR mediate receptor-specific effects, since there are intrinsically different effects of MR and GR activation in the same cell type, presumably via genomic mechanisms (Joëls et al. 1991; Karst et al. 2000). In a recent study, we have directly addressed MR/GR selectivity by comparing genomic binding sites in the rat hippocampus for MR and GR (van Weert et al. 2017). It transpired that in addition to common MR/GR binding sites on the DNA, there are many loci that exclusively bind either GR or MR. In the vicinity of all MR-specific binding sites, the Atoh/NeuroD consensus sequence was found, and this sequence was absent from GR-specific sites. We therefore suggest that other transcription factors play an important role in determining the MR/GR specificity of a GRE. For other nuclear receptors, there are often transcription factors that bind close to the receptor as well, be it as necessary cell type-specific pioneers or as co-binders to the same loci (Krum et al. 2008; Biddie et al. 2011).

Our motif analysis suggests that mainly MR homodimers and to some extent MR–GR heterodimers are associated with NeuroD sites (van Weert et al. 2017). We have observed in the brains of forebrain MR knockout animals that NeuroD still binds to these loci, suggesting that NeuroD facilitates MR binding to DNA, rather than the other way around (Meijer *et al*, unpublished observations). In cultured cells, DNA-bound NeuroD can potentiate both MR and GR-dependent transcription, which suggests that the interaction may be indirect and dependent on tissue-specific proteins in the complex associated with hippocampal MR (van Weert et al. 2017). The fact that MR is associated with NeuroD also has functional implications. NeuroD factors are bHLH proteins that are developmentally important in shaping the exact phenotype of neurons (Fong et al. 2015). MR recruitment to some of the NeuroD binding loci to the DNA suggests that cortisol via MR is involved in determining the neuronal differentiation of pyramidal and granule cells in the hippocampus. This may be relevant during development, but also in adulthood, e.g., in relation to MR-mediated effects enhancing neuronal excitability (Joëls 1997). In conclusion, in hippocampal tissue selectivity of MR/GR binding to DNA can be explained by cis-acting transcription factors that confer the specificity of a GRE sequence.

### Transcriptional Activation: Target Genes and Coregulators

However insightful the genome-wide MR/GR binding data are, only a relatively small fraction of the detected binding sites is associated with active transcription (Vockley et al. 2016). Apparently, gene regulation by MR and GR does not only depend on DNA binding. To regulate transcription, the receptors engage in interactions with coregulators, which determine both the nature and the extent of transcriptional regulation. Coregulators mediate and modulate transcription in a gene and receptor, and even ligand-specific manner (O’Malley 2007; Rogerson et al. 2014; Atucha et al. 2015), and are therefore likely to contribute to the MR/GR specificity of the transcriptional regulation of a gene. The expression and activity of coregulators is cell type specific, and this likely determines which sets of genes are regulated via MR and GR (Zalachoras et al. 2016).

MR and GR have two AF-domains which interact with coregulator proteins. Over 300 coregulators of AF-2 have been identified, but only a subset will interact with a specific nuclear receptor (Broekema et al. 2018). Accordingly, different knockout mice for coregulator genes show a wide spectrum of neuronal and behavioral phenotypes. (Stashi et al. 2013). The cell type-specific coregulator dependence was studied for GR in some detail for two splice variants of steroid receptor coactivator-1 (SRC-1), coded by the *Ncoa1* gene. The SRC-1a and SRC-1e splice variants differ in their stimulation of steroids receptors in a gene (GRE)-specific manner (Kalkhoven et al. 1998). Oligonucleotide based in situ hybridization showed wide expression of SRC-1 in the brain, with a substantial enrichment of SRC-1a in the hypothalamus and anterior pituitary, suggestive of a specific role in the regulation of neuroendocrine axes (Meijer et al. 2000a). Indeed, whole SRC-1 knockout mice show gene-specific GR resistance for the repression of corticotrope POMC expression and hypothalamic CRH expression (Winnay et al. 2006; Lachize et al. 2009). To address the role of the specific splice variants, we employed exon-skipping, in which oligonucleotides bind to primary RNA molecules to interfere with splicing (Zalachoras et al. 2011). The oligonucleotides are taken up very effectively after local injection in the brain. In this way, we were able to show that increasing the ratio of SRC-1A:SRC-1E led to a loss of CRH induction in the central nucleus of the amygdala, whereas regulation of the FKBP5 gene via GR remained intact. Skewing SRC-1 splice variants in the CeA also led to changes in fear memories (Zalachoras et al. 2016). Genome-wide analysis of gene expression in the brain has since shown that as a rule, coregulator expression is brain area specific (Mahfouz et al. 2016), and may therefore be responsible for many cell-specific (transcriptional) effects of corticosteroids.

There seems to be little MR/GR specificity for these AF-2 interacting coactivators (Broekema et al 2018), which perhaps is not surprising given the similarity between the MR and GR ligand-binding domain. The AF-1 domain has been more difficult to study, but likely displays substantially more receptor specificity (Meijer et al. 2005; Fuller et al. 2017). So far, many of the receptor–coregulator interactions have only been studied in vitro and have been validated on a limited number of endogenous genes. As a first approach to studying in vivo interactions between MR/GR and their many (potential) coregulators, it is possible to first evaluate the occurrence in the same cell type using genome-wide expression data for the mouse and human brain (Mahfouz et al. 2016). The availability of more refined (single cell based) datasets (Cembrowski et al. 2016), and analysis of genome-wide coregulator recruitment (Zwart et al. 2011) will help to further understand how MR and GR regulate specific sets of target genes under particular conditions.

## Factors Modifying Genomic Activity

**Fig. 3:**
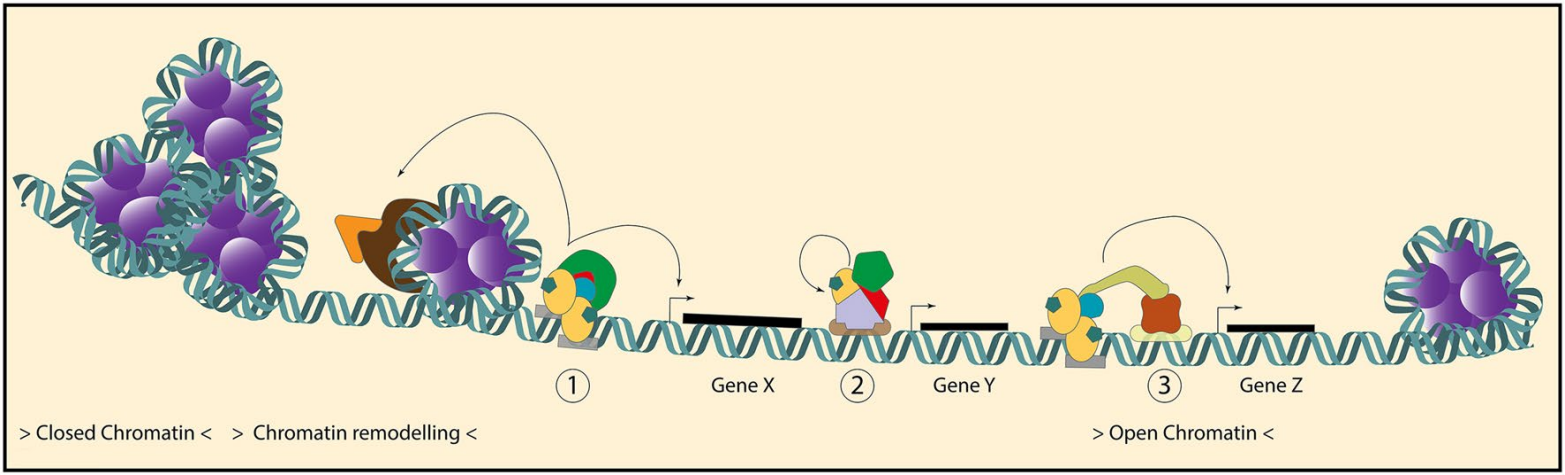
GR and MR interactions on the DNA. A prerequisite for MR/GR binding to its GRE is accessibility of the target sequence in a region of open chromatin. Besides binding to readily available regions of open chromatin, the GR is able to increase accessibility of closed chromatin regions via interactions with chromatin remodeling proteins. The MR and GR can transactivate and repress gene transcription via a number of mechanisms: (1) direct DNA binding to the GRE leads to the recruitment of a specific set of coregulator proteins which together initiate transcription. (2) MR and GR bind to a DNA-bound transcription factor (tethering) and modulate the transcription of the target gene. (3) MR/GR bind to the GRE and interacts with another DNA-bound transcription factor (co-binder) leading to receptor-specific target gene regulation. Of note, the chromatin accessibility and expression of chromatin remodellers/coregulator/co-binders is cell-specific and will contribute to the divergent effects of MR and GR on transcription

In the process that goes from ligand binding to transcriptional modulation, MR and GR engage in several molecular processes that involve numerous potential interacting proteins. In short, proven differences between MR and GR-dependent transcription in the brain occur at the level of DNA-binding sites, mediated at least in part by distinct interaction partners. Rather than via tethering, it seems that MR/GR interactions with other transcription factors occur in cis. Factors like NeuroD (for MR) and NF-1 (for GR) seem to confer binding specificity, and in this way direct MR and GR-specific transcriptional regulation. It is very likely that also receptor-specific coregulator interactions take place, in particular via the poorly conserved AF-1 domain (Fuller et al. 2017). Lastly, we cannot rule out that the well-described differences in tethering partners are also relevant for the brain, in the context of in specific, activated cell populations like astrocytes or microglia under inflammatory conditions. Cell type-specific expression of the receptor (Mahfouz et al. 2016), context-specific post-translational modifications of all interacting partners (Vandevyver et al. 2014), sensitivity to specific ligands, and the temporal variation of hormone availability (Stavreva et al. 2009; Conway-Campbell et al. 2010) are additional layers on top of the intrinsic differences in MR/GR structure (Fig. 3).

Rapid technological developments should resolve a number of current questions on MR and GR-specific signaling. *Omics* approaches like RNAseq and ChIP-seq have been applied at the level of whole hippocampus, or sometimes (mRNA) at microdissected cell populations from hippocampus (Datson et al. 2013). Single-cell sequencing should before long give much more detailed information on transcriptional responses in the whole cellular repertoire of brain structures (Ofengeim et al. 2017). The ATAC-seq approach is very promising, as it can already at this stage probe general chromatin accessibility in as little as 500 neurons (Buenrostro et al. 2013). Large-scale profiling of in vivo protein interaction is possible for particular sets of interactions, such as the coregulators that interact with the GR AF-2 (Desmet et al. 2014), and in open approaches (Lempiäinen et al. 2017).

Given the recent and expected future technological advancements, there will be a veritable *mer à boire* with respect to studies of corticosteroid receptor signaling. These will include applications in different brain structures, possibly from post mortem human samples, in contexts that are relevant either for normal adaptive responses to stressors or to understanding and reversing the contribution of corticosteroids to disease.
